# Exploratory Analysis of Inflammatory Biomarkers and Their Association with Psychological Burden in Mexican Informal Caregivers: A Cross-Sectional Study in the Emergency Department

**DOI:** 10.3390/ijms27073316

**Published:** 2026-04-07

**Authors:** José Juan Gómez-Ramos, Natali Montoya-Mendoza, Ana Miriam Saldaña-Cruz, Sergio Gabriel Gallardo-Moya, Omar Karim López-Barajas, Rafael Chávez-Moreno, Alejandro Marín-Medina

**Affiliations:** 1 Especialidad de Medicina de Urgencias, Adscrita al Centro Universitario de Ciencias de la Salud (CUCS), Universidad de Guadalajara, Guadalajara 44340, Jalisco, Mexico; josejuan79@yahoo.com (J.J.G.-R.); natalimontoya1@gmail.com (N.M.-M.); 2 Departamento de Urgencias, Hospital General de Zona 89, Instituto Mexicano del Seguro Social (IMSS), Guadalajara 44100, Jalisco, Mexico; omarkarimlopezbarajas@gmail.com; 3 Departamento de Fisiología, Instituto de Terapéutica Experimental y Clínica, Centro Universitario de Ciencias de la Salud (CUCS), Universidad de Guadalajara, Guadalajara 44340, Jalisco, Mexico; ana.saldanac@academicos.udg.mx (A.M.S.-C.); sergio.gallardo@alumnos.udg.mx (S.G.G.-M.); 4 Licenciatura en Médico Cirujano y Partero, Centro Universitario de Ciencias de la Salud (CUCS), Universidad de Guadalajara, Guadalajara 44340, Jalisco, Mexico; rafael.chavez8565@alumnos.udg.mx; 5 Departamento de Biología Molecular y Genómicas, Centro Universitario de Ciencias de la Salud (CUCS), Universidad de Guadalajara, Guadalajara 44340, Jalisco, Mexico

**Keywords:** caregivers, inflammation, psychoneuroimmunology, stress, physiological, biomarkers, Interleukin-6, C-reactive protein, tumor necrosis factor-alpha

## Abstract

This cross-sectional exploratory study aimed to explore the association between inflammatory biomarkers and multidimensional psychological burden in informal primary caregivers of dependent older adults in an emergency department. We included 78 caregivers and up to 89 controls. Multidimensional psychological burden (perceived stress, depression, anxiety, and caregiver burden) was assessed using standardized instruments. Morning serum levels of cortisol, interleukin-6 (IL-6), tumor necrosis factor-alpha (TNF-α), and C-reactive Protein, were measured. Statistical analyses included between-group comparisons (Mann–Whitney U test), correlations (Spearman rank correlation coefficient), and hierarchical block regression adjusted for obesity, diabetes, and hypertension in the caregiver group. Multiplicity was addressed using the False Discovery Rate (FDR) procedure, and the findings were validated through 1000 bootstrap resamples. Caregivers had significantly higher levels of TNF-α compared to controls (*p* = 0.021), a finding confirmed by bootstrap analysis (95% CI: −2.6730 to −0.2940). IL-6 levels were positively correlated with trait anxiety (*p* = 0.007) and caregiver burden (*p* = 0.019). Comorbidity-adjusted hierarchical regression confirmed significant associations between IL-6 levels and trait anxiety and caregiver burden (Δ*R*^2^ = 0.123, *p* = 0.007), although these associations did not remain significant after adjustment for FDR. Caregivers showed elevated levels of TNF-α and exploratory associations between IL-6, trait anxiety, and caregiver burden, justifying confirmation in studies with a larger number of participants.

## 1. Introduction

Modern societies are experiencing significant demographic changes, transitioning from a young society to one composed mostly of older adults [1]. In this sense, aging presents a challenge for health services and public health, as the needs and demands for care from an increasingly aging population are growing [2,3], meaning that older adults, at some point in their lives, will require assistance from others to carry out basic activities of daily living related to hygiene, nutrition, and mobility [2].

In most countries, this care is provided by informal caregivers, primarily family members [4]. An informal caregiver is someone (family member or close friend) who provides care, free of charge and without training or professional qualifications, to a loved one who is not self-sufficient and who generally requires long-term care due to functional dependence from advanced age and/or illness. This type of caregiver includes family members, friends, and even neighbors; the term “family caregiver” is often used interchangeably to refer to this type of caregiver [5,6].

In Latin America, informal caregivers provide the main source of care for dependent individuals. Census data from four countries in the region (Brazil, Costa Rica, Ecuador, and Uruguay) indicate that only about 1% of people aged 65 and older live in residential care facilities [7]. In Mexico, families primarily provide care for older adults through informal home-based arrangements. Public care services remain limited, while the private sector offers more developed alternatives; however, the high cost of these services makes them inaccessible to most Mexicans [8]. To better understand elder care and informal caregiving in Mexico, researchers conducted two national surveys: the National Survey of Time Use (ENUT) in 2009 and the Survey of Labor and Social Coresponsibility (ELCOS) in 2012. The results show a growing proportion of individuals aged 60 and older who require long-term care, including assistance with activities of daily living. They also highlight the significant social and economic burden that informal caregiving places on Mexican families, with varying levels of participation among family members [9].

The tension and stress experienced by caregivers affect multiple dimensions, including the biological, psychological, and social spheres [10]. When individuals perceive an acute stressful event, the nervous, cardiovascular, endocrine, and immune systems activate a cascade of physiological changes that together constitute the stress response, which is generally adaptive. Among these processes, researchers have proposed the phenomenon of “inflammaging,” defined as a state of chronic low-grade inflammation that chronic stress may accelerate. In caregivers of older adults, this process may have deleterious consequences and appears to play a relevant role in the neuroendocrine and metabolic alterations associated with prolonged stress [11,12]. Although stress has been proposed to activate pro-inflammatory pathways, studying this relationship remains challenging, particularly in the complex emergency department (ED) setting. Chronic stress and reduced quality of life among caregivers often contribute to burnout, which may increase healthcare utilization, ED use, and the risk of morbidity and mortality [10]. Most previous research has focused on neuroendocrine biomarkers such as cortisol; however, examining additional inflammatory biomarkers may provide a broader understanding of the relationship between inflammation and psychological burden. Existing evidence remains limited and inconsistent, suggesting that these associations may vary depending on contextual, methodological, and individual factors [13,14].

Identifying inflammatory signals in caregivers could contribute to the early detection of risk factors and adverse health outcomes. These findings may help develop more effective interventions and support strategies in long-term care settings, ultimately improving caregivers’ well-being and advancing understanding of the biological mechanisms underlying caregiver health. Several validated instruments assess psychological burden in caregivers, including the Zarit Burden Interview (ZBI) for caregiver burden, the State-Trait Anxiety Inventory (STAI) for anxiety, the Perceived Stress Scale (PSS-14) for perceived stress, and the State–Trait Depression Inventory (STDI) for depression. These tools allow researchers to measure different dimensions of emotional distress, stress, depression and anxiety among caregivers of individuals with chronic illnesses, older adults, and healthcare professionals [15].

In Mexico, evidence on the impact of chronic stress associated with caring for dependent individuals is limited, especially regarding its relationship with inflammatory biomarkers and its potential contribution to morbidity and mortality among informal caregivers. To address this gap, this study explored the association between inflammatory biomarkers and multidimensional psychological burden in primary informal caregivers of dependent older adults admitted to the ED of a secondary-level hospital in western Mexico.

## 2. Results

### 2.1. Sociodemographic Characterization and Functional Status of the Care Recipient

Table 1 presents an exploratory comparison of the sociodemographic characteristics of the 78 informal primary caregivers and the control group. The mean age of caregivers was 51.06 ± 12.61 years, whereas the control group had a mean age of 47.22 ± 9.67 years. The Kolmogorov–Smirnov test indicated that age followed a normal distribution in both groups (*p* > 0.05), and no significant differences were observed between them (*t* = 1.088, *p* = 0.278). Regarding sex distribution, women predominated in both groups, accounting for 55 participants (70.51%) in the caregiver group and 67 (75.28%) in the control group. The chi-square test showed no statistically significant differences in sex distribution between the groups (*χ*^2^ = 0.48, *p* = 0.488). Therefore, the groups were matched in relation to age and sex.

In terms of employment status, 47.44% of caregivers were employed, whereas 29.49% reported being homemakers. Regarding educational attainment, the largest proportion of participants (26.92%) held a bachelor’s degree.

Regarding the relationship to the care recipient, the most common relationship was being a child (58 cases; 74.36%), followed by being a spouse (14.10%). Additionally, 62.82% of caregivers reported at least one comorbidity. The most prevalent conditions were hypertension (32.05%), followed by obesity (23.08%), diabetes mellitus (16.67%), and lipid metabolism disorders (15.38%). Additionally, a considerable proportion of the sample (32.05%) reported a depressive disorder. While clinical comorbidity data (specifically diabetes mellitus, hypertension, and obesity) were not available for the control group, these variables were rigorously accounted for as potential confounders within the caregiver cohort. This was achieved through hierarchical regression models designed to assess the independent contribution of caregiving stress to the inflammatory profile.

In the first block of the hierarchical model, the inclusion of clinical comorbidities (diabetes mellitus, hypertension, and obesity) did not show a statistically significant contribution (*R*^2^ = 0.032; F(3,74) = 0.804, *p* = 0.495), indicating that these factors alone did not explain the variability of IL-6 in this sample. Conversely, the addition of trait anxiety and caregiver burden in the second block resulted in a significant increase in explained variance (*ΔR*^2^ = 0.123; F_change_(2,72) = 5.257, *p* = 0.007). The final adjusted model was statistically significant (F(5,72) = 2.641, *p* = 0.030), explaining 15.5% of the total variance (*R*^2^_adj_ = 0.096).

Among the older adults receiving care, the mean age was 79.31 ± 9.43 years, and 60.26% (*n* = 47) were women. Most older adults (85.90%) lived in the same household as their primary caregiver. The morbidity profile of the care recipients also showed a high prevalence of chronic degenerative diseases, particularly hypertension (75.64%) and diabetes mellitus (52.56%). Additionally, 28.21% had a diagnosis of dementia, and the same proportion (28.21%) required renal replacement therapy. Other relevant conditions included a history of stroke (23.08%) and chronic obstructive pulmonary disease (16.67%).

### 2.2. Dynamics and Burden of Informal Care Provision

Table 2 presents the characteristics of the caregiving dynamics reported by the participants. Caregivers indicated that they devoted an average of 15.46 (±6.43) hours per day to caregiving activities. The mean duration of the caregiving role was 46.27 (±38.83) months.

Regarding the activities performed, participation was almost universal in accompanying care recipients to medical appointments (94.87%), carrying out healthcare-related tasks (93.59%), and assisting with basic activities of daily living (92.31%). A high proportion of caregivers also performed accompaniment and supervision tasks, most commonly providing general companionship (87.18%) and monitoring the patient (78.21%). Other activities, although still reported by the majority of participants, included household tasks, assistance with walking, and support with basic personal hygiene.

### 2.3. Comparative Analysis of Stress and Inflammation Biomarkers Between Caregivers and Control Group

**Figure 1:**
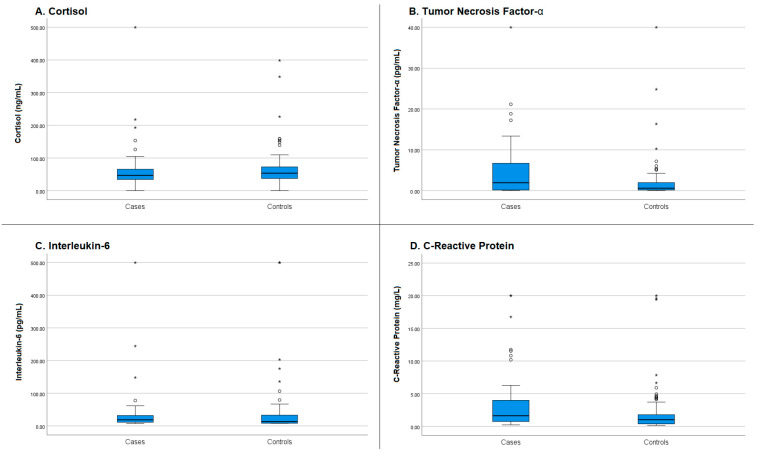
Comparison of stress and inflammation biomarkers between informal caregivers and the control group. (**A**) Cortisol; (**B**) Tumor necrosis factor alpha (TNF-α); (**C**) Interleukin-6 (IL-6); (**D**) C-reactive Protein (CRP). Box plots represent the median (horizontal line), interquartile range (box), and minimum and maximum values (whiskers). Individual points (circles and asterisks) represent moderate and extreme outliers, respectively, indicating data points that fall beyond the whiskers of the distribution. Although both TNF-α; (**B**) and CRP (**D**) showed statistically significant differences (*p* < 0.05) in the initial comparison, only TNF-α maintained statistical robustness after bootstrap analysis (BCa CI did not cross zero).

Table 3 presents the comparison of biomarker levels between the primary caregiver group and the control group. Regarding inflammatory markers, the exploratory analysis identified differences in two biomarkers. TNF-α levels were significantly higher in caregivers, with a median of 1.95 pg/mL (IQR: 0.17–6.74), compared with 0.62 pg/mL (IQR: 0.17–1.99) in the control group (*p* = 0.021). The robustness of this finding was supported by bootstrap analysis, which showed a significant median difference (BCa CI: −2.6730 to −0.2940). Similarly, CRP levels were higher in caregivers, with a median of 1.63 mg/L (IQR: 0.73–3.99), compared with 1.01 mg/L (IQR: 0.37–1.87) in the control group (*p* = 0.002) (Figure 1). However, bootstrap analysis did not confirm the robustness of this difference (BCa CI: −1.4030 to 0.0802), suggesting that this finding should be interpreted with caution.

In contrast, no statistically significant differences were found in serum cortisol levels (*p* = 0.149), with medians of 46.99 ng/mL for caregivers and 53.65 ng/mL for the control group. Similarly, IL-6 showed no significant variation between groups (*p* = 0.135), with medians of 18.73 pg/mL and 13.37 pg/mL, respectively.

### 2.4. Mental Health Assessment: Stress, Burden, Anxiety and Depression in Caregivers

Table 4 details the results of the psychometric instruments applied to the 78 informal primary caregivers. The average scores were a PSS-14 of 27.79 (±6.72) and a ZBI of 35.12 (±13.26) points; in the distribution by categories, 79.49% were classified as “no burden”, while 15.38% presented a mild burden and 5.13% a severe burden.

In terms of emotional health, the STAI total anxiety score was 90.54 (±12.29). When analyzing by subscales, 67.95% of caregivers exhibited high levels of trait anxiety (STAI-T), whereas the predominant level for state anxiety (STAI-S) was medium (55.13%), followed by high (39.74%). Finally, the STDI total score was 98.47 (±6.65). Notably, in the Trait Depression (STDI-T) subscale, 97.44% of caregivers scored at a high level. In the State Depression (STDI-S) assessment, the distribution was more balanced, with 48.71% at a high level and 41.02% at a medium level.

### 2.5. Exploratory Correlation Analysis Between Psychological Burden and Biomarkers

Regarding the correlation analysis between serum biomarkers and psychological scales (Table 5), the associations were validated using 95% BCa CI. A significant positive correlation was identified between IL-6 levels and trait anxiety (STAI-T) (*rho* = 0.305, *p* = 0.007, BCa CI: 0.105 to 0.473) and with caregiver burden (ZBI) (*rho* = 0.265, *p* = 0.019, BCa CI: 0.050 to 0.459). In both cases, the statistical significance was confirmed by the fact that the BCa CI did not cross the zero (0) threshold, indicating that these relationships remain stable across 1000 bootstrap resamples.

In contrast, although the association between IL-6 and STDI-S showed a nominal *p*-value of 0.049, it was considered non-significant as its BCa CI crossed zero (−0.459 to 0.029), demonstrating a lack of robust evidence for this link. Furthermore, IL-6 showed no significant association with any other psychometric scales, including PSS-14, STAI-S, or STDI-T. Similarly, correlations for cortisol, TNF-α, and CRP remained non-significant across all psychological dimensions assessed.

The reliability of all reported correlation coefficients was supported by low Bootstrap Standard Errors (SE), which consistently ranged between 0.09 and 0.12, confirming high stability and convergence in the estimation of the biological-psychometric associations.

Finally, to further address the risk of false positives (FDR) from multiple comparisons (*n* = 24), the Benjamini–Hochberg procedure was applied. While the adjusted thresholds were not reached, both nominal significance (*p* < 0.05) and the stable BCa intervals were reported, adopting a conservative stance in the interpretation of these exploratory findings.

**Figure 2:**
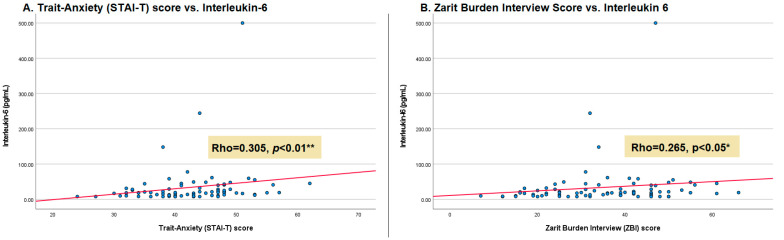
Correlation between psychological burden and inflammatory response in informal primary caregivers. The panel shows the Spearman correlation between serum IL-6 levels (pg/mL) and: (**A**) the STAI (STAI-T); (**B**) the ZBI.

The correlation between the inflammatory response, affective symptoms, and burden is illustrated in Figure 2. A positive correlation can be observed between trait anxiety and caregiver burden, identifying that caregivers with higher scores on these scales also exhibited higher concentrations of IL-6.

### 2.6. Exploratory Biomarker Analysis by Caregiver Burden Levels

Finally, a comparative analysis of biomarkers was performed after categorizing the group of caregivers according to the presence or absence of burden using the ZBI (Table 6). For this exploratory subgroup analysis, subjects with mild and severe burden were grouped into a single category (with burden, *n* = 16) to compare them with those without burden (*n* = 62).

No statistically significant differences were observed in biomarker levels between the two groups. However, a nominal trend toward higher IL-6 levels was observed in the group with caregiver burden, with a median of 23.78 pg/mL compared to 18.14 pg/mL in the group without burden.

Similarly, CRP showed medians of 2.85 mg/L in caregivers with burden and 1.55 mg/L in those without (*p* = 0.496). Regarding cortisol and TNF-α, no significant associations linked to the presence of burden were identified in this sample.

## 3. Discussion

Analyzing the sociodemographic characteristics of our study population, we identified that the predominant profile is that of a woman, with a mean age of 51, a bachelor’s degree, and employment, whose primary relationship to the care recipient is filial (daughter). This profile aligns with national trends reported by INEGI [16] and INAPAM [17], which indicate that informal care in Mexico is primarily provided by women between the ages of 39 and 59, 56.3% of whom combine caregiving with paid employment. Socially, care provision in Mexico continues to be based on a moral norm of reciprocity and affection within the family unit.

In our study, TNF-α was the only biomarker that showed a significant elevation in caregivers compared to the control group (1.95 pg/mL vs. 0.62 pg/mL, *p* = 0.021). Regarding the neuroendocrine profile, caregivers showed a non-significant trend toward lower cortisol levels (46.99 ng/mL) compared to controls (53.65 ng/mL; *p* = 0.149). While some models of chronic stress suggest that prolonged exposure can lead to complex adaptations of the Hypothalamic–Pituitary–Adrenal (HPA) axis, including flattened diurnal profiles or altered baseline levels [18,19,20], our findings did not provide evidence of a significant hypocortisolism state in this cohort. In this exploratory cohort, no significant associations were observed between baseline cortisol levels and the psychometric scales assessed. While some studies have reported links between significantly low cortisol levels and psychological distress (specifically in populations with obsessive-compulsive symptoms or severe anxiety) [21], our findings do not suggest a state of clinically significant HPA-axis dysfunction in this caregiver sample. The absence of a correlation between cortisol and the psychological dimensions measured (ZBI, STAI, PSS-14) further underscores the complexity and multifaceted nature of the neuroendocrine stress response. These results suggest that in this clinical setting, baseline morning cortisol may not be a sensitive marker for individual variations in perceived burden or anxiety levels.

Prolonged stress has been reported to be associated with a systemic pro-inflammatory state and a persistent sympathetic nervous system response that exerts a positive regulatory effect on certain immune response genes and activates macrophages and T cells, thereby increasing the secretion of pro-inflammatory cytokines [20,22]. Our exploratory analysis revealed a significant difference in TNF-α levels between caregivers and controls. In this regard, caregivers of allogeneic hematopoietic stem cell transplant patients who dedicate many hours to their care have been reported to have higher levels of pro-inflammatory cytokines, including TNF-α [23]. This cytokine has been reported to be crucial for communication between the immune system and the brain, making further exploration of its potential role in chronic caregiving stress necessary [24].

In the case of CRP, the initial analysis revealed a significant difference between caregivers and controls. However, bootstrap analysis did not confirm the robustness of this finding, suggesting that the result should be interpreted with caution. A systematic review identified CRP as one of the most variable biomarkers in studies assessing associations between stressors and inflammatory responses across different contexts, indicating that this marker may exhibit dynamic behavior under stressful conditions [25]. In this regard, Von Känel et al. [26] reported a significant reduction in CRP levels following the death of the care recipient. This observation suggests that elevated CRP levels in caregivers may reflect the direct impact of an active stressor rather than a static inflammatory state associated with caregiving itself [27]. This chronic biological and psychological burden among caregivers also has important implications for the healthcare system, as suggested by Mausbach et al. [28]. In their study, the authors annually assessed depressive symptoms and ED visits and found that higher levels of caregiver stress significantly predicted the frequency of ED visits and hospitalizations (HR = 1.20). In this context, caregivers in our population, who reported high levels of stress while dedicating an average of 15.4 h per day to caregiving, may face not only an increased risk to their mental health but also a potential deterioration of their physical health. Consequently, this situation may contribute to a greater demand for emergency medical services.

In our study, IL-6 levels showed a significant correlation with the STAI-T and ZBI scales. A similar association was reported by Bidwell et al. [29], who found that caregiver anxiety (*β* = 0.35, *p* = 0.003) was a significant predictor of systemic inflammation. However, our exploratory analysis did not identify a correlation with state or trait depression, despite the fact that nearly the entire sample (97.44%) presented medium to high levels on the STDI scale. This discrepancy may be related to the highly pleiotropic nature of IL-6, a biomarker that is sensitive to multiple modulating factors not controlled for in this study, which may obscure potential dose–response relationships in chronic affective disorders [27]. From this perspective, Aw et al. [30] recently conducted a longitudinal study with a 9-year cohort to assess IL-6 levels and their interaction with psychosocial factors. Their findings suggested that IL-6 may act as a biological moderator that amplifies the impact of social stress on future depressive symptoms (*β* = 0.08, *p* < 0.01). While our cross-sectional design does not allow us to establish temporal or causal relationships, the observed associations between IL-6 and caregiving burden in our sample could suggest a similar process of biological sensitization in caregivers and contribute to the persistence of stress-related traits over time, warranting further investigation with a larger sample size. In our exploratory study, we found no statistically significant differences in biomarker levels between caregivers with and without burden. Although caregivers with burden showed higher median levels of IL-6 and CRP, these differences did not reach statistical significance. Previous studies have similarly reported a lack of correlation between perceived extreme caregiver burden and neuroendocrine or inflammatory responses [31,32]. In the case of IL-6, research suggests that significant stress-related associations tend to emerge only when additional factors are present, such as low self-efficacy or specific psychiatric comorbidities [33].

Our study had several limitations that should be considered. First, its cross-sectional, single-center design prevents us from establishing causal relationships and limits the generalizability of the findings to other contexts and populations. Second, variability in sample availability within the control group led to fluctuations in sample size, which should be taken into account when assessing the consistency of comparisons. Furthermore, we measured biomarkers (including cortisol) only once in the morning, which prevents a comprehensive assessment of the dynamic nature of caregiver stress and the circadian rhythm of the HPA axis. We also had difficulty achieving optimal matching in the control group for comorbidities (overweight/obesity, diabetes, and hypertension) due to the high prevalence of these conditions in our population and the significant public health challenges they pose. Since we did not make analytical adjustments for these comorbidities, the comparisons and associations presented here should be interpreted as exploratory. Further longitudinal studies with appropriately adjusted models are needed to assess the progression of the observed pro-inflammatory signals and their possible relationship to the long-term inflammatory process associated with chronic caregiver stress. Furthermore, future studies should incorporate dynamic measurements of the circadian rhythm of cortisol to more accurately characterize HPA axis activity and clarify its potential role in the chronic stress experienced by caregivers. Finally, it is important to investigate whether cognitive-behavioral interventions focused on resilience and self-efficacy can modulate the immune response in caregivers.

## 4. Materials and Methods

### 4.1. Study Design, Settings, and Participants

This was a comparative exploratory study with a cross-sectional design. It was conducted in the ED of the Hospital General de Zona No. 89 (HGZ 89) of the Instituto Mexicano del Seguro Social (IMSS), in Guadalajara, a secondary-level hospital located in western Mexico. The hospital has 226 inpatient beds and 137 outpatient beds and provides approximately 50,000 emergency consultations annually.

### 4.2. Selection of Participants

From February to July 2025, a total of 167 people were recruited and divided into two study groups. One group consisted of caregivers (referred to as cases) who met the following inclusion criteria: individuals who were informal primary caregivers of dependent older adults aged 65 and over hospitalized in the ED and who agreed to participate in the study and signed the informed consent form. The comparison group (referred to as the control group) consisted of individuals who were not caregivers and were recruited using a convenience sampling strategy from the common areas of the same medical unit or outpatient clinic. For both groups, people with a history or recent use of glucocorticoids, smoking, a diagnosis of rheumatic or inflammatory diseases, a diagnosis of cancer, pregnancy, a history of contraceptive use, recent bacterial or viral infections, or a diagnosis of HIV were excluded. However, given the clinical context, strict matching was not achieved for other inflammatory confounding factors such as obesity/overweight, hypertension, or diabetes.

The sample size for the case group was calculated using Cochran’s formula for finite populations [34]. The population size (*N*) was defined as 94,920 older adults aged 65 years or older assigned to the Área Médica de Gestión Descentralizada No. 1402, Zona 11 Chapultepec, who are potential users of the ED and may require a caregiver. A 95% confidence level (*Z*) was assumed. The expected prevalence of the event (*p*) was set at 5%, based on previously reported levels of caregiver burden in studies conducted in Mexican populations in comparable contexts, which range from 5% to 26% [35,36,37,38,39].

For this estimate, we considered the prevalence reported by Vega-Silva et al. [35] in 2023 among caregivers of individuals with diabetes-related complications, assessed using the ZBI as a diagnostic measure and conducted within the IMSS setting. The calculated sample size was 73 informal primary caregivers. To account for potential data loss during collection or possible issues during the processing of biological samples, the final sample size was increased to 78 participants.

A convenience sampling strategy was used to recruit participants. This sample size was intended to provide a preliminary exploration of the variables of interest. Furthermore, for comparative analysis, a control group of 89 non-caregivers was formed. A 1:1.14 ratio between caregivers and non-caregivers was established to maximize the statistical power of the non-parametric tests. The final number of subjects within the control group varied across biomarkers: cortisol (*n* = 83), TNF-α (*n* = 71), IL-6 (*n* = 70), and CRP (*n* = 89). This variability was primarily determined by the availability of serum volume and the accessibility of specific reagents; CRP and cortisol were characterized by higher reagent accessibility and greater serum availability, while the sample sizes for IL-6 and TNF-α were constrained by limited reagent availability and occasional losses during the specialized processing of these high-sensitivity biomarkers. Due to this variability in sample sizes across biomarkers, the comparisons are considered exploratory. All participants provided written informed consent.

### 4.3. Data Collection

Information was collected on the caregivers’ sociodemographic characteristics, including age, sex, employment status, comorbidities, educational level, marital status, and relationship to the care recipient. Data were also obtained on the characteristics of the older adults receiving care, including age, sex, comorbidities, sensory and mobility impairments, tobacco and alcohol use, place of residence, and level of dependency. In addition, care-related variables were recorded, such as the number of hours per day dedicated to caregiving, total time spent providing care, and the types of activities performed. This information was used to characterize the baseline profile of the sample.

Affective state and perceived stress were assessed using the PSS-14, ZBI, STDI, and STAI scales. Finally, morning levels of cortisol, IL-6, TNF-α, and CRP were measured. Due to the exploratory nature of the study, these clinical and sociodemographic variables were recorded for descriptive purposes but were not included as covariates in the primary comparative analyses. The database for this article is available as supplementary material.

#### 4.3.1. Assessment of Functional Dependency

The dependency status of hospitalized older adults was assessed using the Katz Index (KI) [40], which evaluates performance in six basic activities of daily living: eating, bathing, dressing, toileting, transferring, and urinary continence. The instrument classifies functional status into eight categories (A–G, with an additional category O) and assigns a score ranging from 0 (total dependence) to 6 (full independence) [40,41,42,43,44].

The KI has demonstrated good reliability for predicting functional outcomes over time in older adults receiving short-term care, hospitalized patients, and individuals who have experienced stroke, with reported reliability coefficients ranging from 0.87 to 0.94 [37,38,39,40,41,42]. The instrument has also been validated in its Spanish version [41,42]. Its use is widely recommended in the Mexican clinical context, and it is the reference instrument included in the National Clinical Practice Guidelines for geriatric assessment in Mexico [45].

Scores are interpreted according to the level of functional dependence, with 6 indicating full function, 4 moderate impairments, and 2 or less severe functional impairment [21,41,43]. For the purposes of this exploratory study, older adults with a KI score of 4 or less were classified as dependent in order to characterize the caregiving demands of the participants.

#### 4.3.2. Collection, Storage and Processing of Samples

Blood samples from both groups were collected Monday through Friday between 7:00 and 9:00 a.m. after an overnight fast. A single 5 mL blood sample was obtained by venipuncture following the standardized procedures of the Clinical Laboratory Department of HGZ 89 and collected in polypropylene Vacutainer^®^ tubes without anticoagulant. The tubes were left at room temperature to allow clot formation, after which serum was separated by centrifugation at 3000 g for 15 min. During processing, samples were kept in sealed containers to prevent contamination or evaporation. Serum aliquots were stored at −20 °C and transported in a refrigerated container (2–8 °C) to the laboratory of the Institute of Experimental and Clinical Therapeutics at the University Center of the Universidad de Guadalajara, where they were stored at −20 °C until analysis.

#### 4.3.3. Determination of Biomarker Levels

IL-6, TNF-α, CRP, and cortisol levels were determined in serum under the following conditions:

- Serum IL-6 levels were determined using a sandwich enzyme-linked immuno-sorbent assay (ELISA) with the commercial LEGEND MAX™ High Sensitivity Human IL-6 kit (BioLegend, San Diego, CA, USA, Cat. No. 430507). This assay has a detection limit of 0.16 pg/mL and a quantification range of 7.8–500 pg/mL. The procedure was performed strictly according to the manufacturer’s instructions.
- For the determination of TNF-α, the LEGEND MAX™ High Sensitivity Human TNF-α kit (BioLegend, Cat. No. 430217) was used, which has a minimum detection limit of 0.190 ± 0.092 pg/mL and a quantification range of 0.625–40 pg/mL. As with IL-6, the sandwich ELISA technique was used following the manufacturer’s instructions.
- Serum cortisol levels were quantified using the ELISA technique with the Calbiotech Cortisol ELISA kit (Calbiotech, El Cajon, CA, USA, Distributor MEXLAB, Cat. 6001013). The assay has a detection limit of 1.5 ng/mL and a reference range of 50 to 230 ng/mL. The procedure was performed according to the manufacturer’s instructions.
- Serum levels of high-sensitivity C-reactive Protein (hs-CRP) were determined using fixed-time immunonephelometry. The commercially available NEPHSTAR^®^ hsCRP kit (Goldsite Diagnostics Inc., Shenzhen, China, Cat. No. DK025) was used on a NEPHSTAR^®^ protein analyzer. The procedure was performed strictly according to the manufacturer’s specifications, considering a reference range of 1–2 mg/L.

#### 4.3.4. Assessment of Affective State and Perceived Stress

After the sample was taken, the perceived levels of stress and burden and the existing symptoms of anxiety and depression were evaluated through the following validated scales:

- ZBI: This is a self-reporting instrument that assesses the burden of informal primary caregivers by identifying the common feelings experienced by those who care for another person. It consists of 22 Likert-type items with five response levels: 1 = never, 2 = rarely, 3 = sometimes, 4 = often, and 5 = always [46]; the total score ranges from 22 to 110; a score below 47 indicates that the person is not burdened; scores between 47 and 55 indicate mild burden, while scores above 55 indicate severe burden [47,48,49].
- To assess perceived stress, the PSS-14 was used. This scale is a self-report instrument that assesses the level of perceived stress during the past month and consists of 14 items with a 5-point response scale (0 = never, 1 = almost never, 2 = occasionally, 3 = frequently, 4 = very frequently). The total PSS-14 score is obtained by reversing the scores of items 4, 5, 6, 7, 9, 10, and 13 (as follows: 0 = 4, 1 = 3, 2 = 2, 3 = 1, and 4 = 0) and then summing the scores of the 14 items. A higher score indicates a greater level of perceived stress. The scale items are easy to understand, and the response options are easy to score [50,51,52].
- STAI: This inventory is designed to assess two independent concepts of anxiety related to time: state (STAI-S; right now, or at this moment) and trait (STAI-T; most of the time) [53,54,55]. The survey is self-administered. Scores on state items range from 0 to 3, with operational criteria based on intensity (0, none; 1, some; 2, quite a lot; 3, a lot). For some state anxiety items, it is necessary to reverse the intensity score (3, none; 2, some; 1, quite a lot; 0, a lot). Items requiring reversal are 1, 2, 5, 8, 10, 11, 15, 16, 19, and 20. While the score for trait items also ranges from 0 to 3, in this case it is based on frequency of occurrence (0, almost never; 1, sometimes; 2, often; 3, almost always). For some trait anxiety items, it is necessary to reverse the score assigned to frequency of occurrence (3, almost never; 2, sometimes; 1, often; 0, almost always); these items are 21, 26, 27, 30, 33, 36, and 39. The total score for each concept ranges from 0 to 60 points. Its interpretation is based on validation performed in a Latin American population. Its interpretation is based on the validation carried out in the Latino population. Both the STAI-T and the STAI-S show the same cutoff points: low ≤ 30 points, medium 30–44 points, and high ≥ 45 points [56,57]. In this study, these scales were used to explore associations between anxiety as a stable personality characteristic (trait) versus a transient emotional state (state) and inflammatory markers.
- STDI: This instrument measures the degree of state depression (STDI-S) and the degree of personal depression as a trait (STDI-T) [58,59]. It consists of 22 items with responses ranging from 1 to 4. Half of these items are positive in terms of depressive states, while the other half are antagonistic to depression. The subject selects the option that best describes their current state from four response choices: not at all (1 point), a little (2 points), quite a bit (3 points), and very much (4 points). The trait depression scale identifies patients prone to depressive states. It also consists of 22 items, with responses ranging from 1 to 4 points: almost never (1 point), sometimes (2 points), frequently (3 points), and almost always (4 points). Scores range from 20 to 80 points for the state scale and from 20 to 88 points for the trait scale. To calculate the score, the sum of the positive depression items and the sum of the negative depression items must be obtained. The difference between these two subtotals is then increased by 50, a value obtained from the statistical analyses performed during the instrument’s construction, in order to standardize the scores. Its interpretation is based on the validation carried out in a Latin American population. The established cut-off points are as follows: in the case of STDI-S, it is considered low ≤ 34 points, medium 35–42 points, and high ≥ 43 points, while for STDI-T it is considered low ≤ 35 points, medium 36–46 points, and high ≥ 47 points [59]. Following the same exploratory approach as with the STAI, the inclusion of both STDI subscales allowed us to examine whether inflammatory signals in caregivers are linked to an immediate depressive state or to a more stable predisposition toward depressive symptoms.

### 4.4. Statistical Analysis

The collected data were initially processed in a Microsoft Excel database (version 2312, belonging to the Microsoft Office LTSC Professional Plus 2021 package), and subsequently, advanced statistical analysis was carried out using IBM SPSS Statistics software (version 27.0), except for the bootstrapping procedure used to calculate 95% BCa CIs for median differences in biomarker intergroup comparisons, which was performed using RStudio (version 4.5.2).

Given the multidimensional nature of the inflammatory response, a phased analysis was performed. First, a descriptive and distributional characterization of the sample was conducted. In the second stage, biomarker levels were compared between groups (cases vs. controls). Subsequently, an exploratory correlation analysis was carried out to identify specific associations between the psychometric scales and the biomarkers within the caregiver group. Finally, a hierarchical regression analysis was implemented to adjust for clinical confounders exclusively within the caregiver group.

Descriptive analysis was performed to characterize the sample. Qualitative variables were summarized using frequencies and percentages, whereas quantitative variables were described using measures of central tendency (mean or median) and dispersion (standard deviation or interquartile range), according to the distribution of each variable. The distribution of continuous variables was assessed using the Kolmogorov–Smirnov test to guide the selection of appropriate statistical tests. As several variables showed a non-normal distribution, non-parametric tests were applied.

For inferential analysis, biomarker levels were compared between the case and control groups using the Mann–Whitney U test. While the relationships between serum biomarker and psychometric scales were evaluated via Spearman’s rank correlation (*rho*). To account for multiple testing inherent to the exploratory design and to reduce the risk of Type I error, *p*-values were adjusted using the Benjamini–Hochberg procedure (false discovery rate, FDR). To ensure the robustness and stability of these findings (particularly given the group size variability), a bootstrap procedure with 1000 resamples was performed for all inferential tests. Statistical significance was established at a nominal *p*-value < 0.05 (FDR-adjusted, where applicable) and further validated when the 95% BCa confidence intervals did not cross the zero threshold. Finally, the precision and convergence of these bootstrap estimates were verified using the Monte Carlo test for median differences and bootstrap Standard Errors for the correlation coefficients.

To control for the effect of clinical inflammatory confounders (diabetes mellitus, systemic arterial hypertension, and obesity) on IL-6 levels, a block-wise (hierarchical) multiple linear regression analysis was performed exclusively within the caregiver group, as clinical comorbidity data were only available for this group. In the first block, comorbidities were introduced to isolate their influence, while in the second block, the psychometric variables of interest were included to assess the unique contribution of psychological stress beyond pre-existing chronic conditions. Although initial linearity and homoscedasticity, independence and criteria were met, the Kolmogorov–Smirnov test indicated a violation of residual normality (*p* < 0.05). To correct the distributional bias and stabilize errors, a logarithmic transformation (log10) of the dependent variable was performed. The final model satisfied assumptions, showing normal residuals (*p* = 0.200), independence of errors (Durbin-Watson = 1.685), and absence of multicollinearity (VIF < 1.5).

## 5. Conclusions

The profile of caregivers in our population is predominantly female and characterized by a high workload associated with caregiving. In this exploratory study, we observed potential inflammatory markers among caregivers, including higher levels of TNF-α and associations between IL-6, trait anxiety, and caregiver burden. Although these associations appeared to be independent of comorbidities, they did not remain significant after correction for multiple comparisons. Therefore, larger studies with adjusted cohort designs are needed to confirm these findings.

Furthermore, the high prevalence of comorbidities highlights the vulnerability of this population and underscores the importance of long-term monitoring of their health status. To better understand the biological impact of caregiving stress, incorporating mental health assessments for informal caregivers of hospitalized patients in ED may facilitate the early detection of psychological distress and the implementation of preventive interventions. Likewise, strengthening support systems that acknowledge the “double burden” often experienced by women—including respite services and financial support—could represent an important component of a comprehensive strategy to protect caregiver health.

## Figures and Tables

**Table 1:** Sociodemographic characteristics of the informal primary caregivers.

Variable	Caregivers (*n* = 78)	Controls (*n* = 89)	*p*-Value
Age	51.06 (±12.61)	47.22 (±9.67)	0.278
Sex			
Men	23 (29.49)	22 (24.72)	0.488
Women	55 (70.51)	67 (75.28)	
Employment status		-	-
Employed	37 (47.44)		
Retired	10 (12.82)		
Unemployed	6 (7.69)		
Homemaker	23 (29.49)		
Student	2 (2.56)		
Educational Attainment			
No formal education	0 (0.00)		
Literate (can read and write)	0 (0.00)		
Elementary school	13 (16.67)		
Middle school	18 (23.08)	-	-
High school	15 (19.23)		
Technical degree	10 (12.82)		
Bachelor’s degree	21 (26.92)		
Graduate degree	1 (1.28)		
Relationship to the care recipient			
Spouse	11 (14.10)		
Child	58 (74.36)		
Sibling	2 (2.56)	N/A	N/A
Other relative	7 (8.97)		
Neighbor	0 (0.00)		
Friend	0 (0.00)		
Presence of at least one comorbidity	49 (62.82)	-	-
Chronic Ischemic Heart Disease	2 (2.56)		
Systemic Arterial Hypertension	25 (32.05)		
Depressive Disorder	6 (7.69)		
Obesity	18 (23.08)		
Diabetes Mellitus	13 (16.67)		
Dyslipidemia	12 (15.38)		

Note. Values are reported as *n* (%) for categorical variables and mean ± standard deviation (SD) for continuous variables. Normality was assessed using the Kolmogorov–Smirnov test with Lilliefors correction. The *p*-value for age corresponds to the intergroup homogeneity test performed via the independent samples *t*-test. The *p*-value for sex corresponds to the intergroup homogeneity test calculated using Pearson’s chi-square test. N/A: not applicable.

**Table 2:** Characteristics of the dynamics of the informal primary caregiver in the provision of care.

Variable	Value (%, ±SD)
Hours of the day dedicated to care	15.46 (±6.43)
Time spent on care (months)	46.27 (±38.83)
Care-related activities	
Basic activities of daily living	72 (92.31)
Accompanying to medical appointments	74 (94.87)
Healthcare-related activities	73 (93.59)
Companionship	68 (87.18)
Basic personal hygiene activities	50 (64.10)
Household chores	59 (75.64)
Walking assistance	51 (65.38)
“Patient monitoring”	61 (78.21)

Note. Values are reported as *n* (%) for categorical variables and mean ± standard deviation (SD) for continuous variables.

**Table 3:** Comparison of stress and inflammatory biomarker levels between groups.

Biomarker	Group	*N*	Median (IQR)	Median Difference (95% BCa CI)	*p*-Value
Cortisol (ng/mL)	Caregivers	78	46.99 (33.93–66.16)	6.655 (−3.6622 to 13.7050)	0.149 ^a^
	Controls	83	53.65 (37.33–74.19)		
TNF-α (pg/mL)	Caregivers	78	1.95 (0.17–6.74)	**−1.33 (−2.6730 to −0.2940)**	**0.021 ^a^**
	Controls	71	0.62 (0.17–1.99)		
IL-6 (pg/mL)	Caregivers	78	18.73 (11.09–33.86)	−5.355 (−8.8008 to 0.1429)	0.135 ^a^
	Controls	70	13.37 (8.16–33.38)		
CRP (mg/L)	Caregivers	78	1.63 (0.73–3.99)	−0.616 (−1.4030 to 0.0802)	**0.002 ^a^**
	Controls	89	1.01 (0.37–1.87)		

Note. Values are presented as median (interquartile range: 25th–75th percentile). The sample size of the caregiver group was kept constant (*n* = 78). The variability in the number of subjects in the control group (*n* between 70 and 89) was due solely to the availability of sufficient serum volume and the technical feasibility of the samples for processing each biomarker. Statistical comparisons between groups were performed using the non-parametric Mann–Whitney U test for independent samples. Significant values (*p* < 0.05) are shown in bold. To increase the statistical certainty of the results despite the variability in the control group sizes, 95% CIs of BCa for the median difference were calculated using 1000 bootstrap resamples. The statistical significance of the BCa interval is confirmed when the range does not cross zero (0), indicating a stable and reliable difference between groups beyond the null hypothesis. ^a^: Calculated using the Mann–Whitney U test for independent samples. Abbreviations: TNF-α, Tumor necrosis factor alpha; IL-6, Interleukin-6; CRP, C-reactive Protein; BCa, Bias-Corrected and accelerated; CI, Confidence Interval; IQR, Interquartile Range; ng/mL, nanograms per milliliter; pg/mL, picograms per milliliter; mg/L, milligrams per liter.

**Table 4:** Mental health assessment, levels of burden, anxiety and depression in informal primary caregivers.

Variable	Value (%, ±SD)
Perceived Stress (PSS-14)	27.79 (±6.72)
Caregiver burden (ZBI)	35.12 (±13.26)
No burden	62 (79.49)
Mild burden	12 (15.38)
Severe burden	4 (5.13)
Total Anxiety Score (STAI)	90.54 (±12.29)
STAI-Trait Anxiety	42.45 (±7.33)
Low	0 (0.00)
Medium	25 (32.05)
High	53 (67.95)
STAI-State Anxiety	48.09 (±6.36)
Low	4 (5.13)
Medium	43 (55.13)
High	31 (39.74)
Total Depression Score (STDI)	98.47 (±6.65)
STDI-Trait Depression	45.88 (±8.95)
Low	0 (0.00)
Medium	2 (2.56)
High	76 (97.44)
STDI-State Depression	52.59 (±5.51)
Low	8 (10.25)
Medium	32 (41.02)
High	38 (48.71)

Note. Values are reported as *n* (%) for categorical variables and mean ± standard deviation (SD) for continuous variables. Abbreviations: PSS-14: Perceived Stress Scale, 14 items; STAI: State-Trait Anxiety Inventory; STDI: State-Trait Depression Inventory; ZBI, Zarit Burden Interview.

**Table 5:** Correlation between mental health scales and stress biomarkers in informal primary caregivers.

Scale/Biomarker	Cortisol	IL-6	TNF-α	CRP
ZBI	−0.084 (0.463 [−0.327 to 0.170])	**0.265** **(0.019 [0.050 to 0.459])**	−0.149 (0.194 [−0.36 to 0.079])	0.140 (0.221 [−0.106 to 0.366])
PSS-14	−0.118 (0.305 [−0.345 to 0.106])	0.184 (0.107 [−0.026 to 0.392)]	−0.050 (0.663 [−0.271 to 0.181])	−0.077 (0.505 [−0.307 to 0.177])
STAI-S	−0.062 (0.489 [−0.283 to 0.174])	0.173 (0.130 [−0.031 to 0.361])	0.043 (0.711 [−0.168 to 0.253])	0.011 (0.922 [−0.215 to 0.220])
STAI-T	−0.173 (0.130 [−0.394 to 0.082])	**0.305** **(0.007 [0.105 to 0.473])**	−0.032 (0.78 [−0.259 to 0.194])	0.031 (0.786 [−0.208 to 0.246])
STDI-S	−0.021 (0.857 [−0.256 to 0.214])	**−0.224** (**0.049** [−0.459 to 0.029])	−0.154 (0.177 [−0.396 to 0.086])	−0.075 (0.517 [−0.292 to 0.137])
STDI-T	−0.121 (0.292 [−0.345 to 0.119])	0.139 (0.224 [−0.047 to 0.329)]	0.028 (0.81 [−0.202 to 0.252])	0.20 (0.079 [−0.079 to 0.457])

Note. Data for each cell are presented as Spearman’s rank correlation coefficient (*rho*); followed by (*p*-value [95% BCa CI]) in parentheses. Significant values are shown in bold. To minimize the risk of Type I error and ensure the robustness of the findings, 95% BCa CIs were calculated using 1000 bootstrap resamples for all associations. Significant correlations where the confidence interval does not cross zero (0) are considered robust evidence of a biological association. To address the multiplicity problem (*n* = 24 comparisons), the Benjamini–Hochberg procedure was applied, providing a conservative framework for the interpretation of these exploratory findings. Abbreviations: ZBI, Zarit Burden Interview; PSS-14, Perceived Stress Scale, 14-item version; STAI-T, State-Trait Anxiety Inventory-Trait; STDI-T, State-Trait Depression Inventory-Trait; STAI-S, State-Trait Anxiety Inventory-State, STDI-S, State-Trait Depression Inventory-State; TNF-α, Tumor necrosis factor alpha; IL-6, Interleukin-6; CRP, C-reactive Protein.

**Table 6:** Comparison of stress biomarker levels according to the presence or absence of caregiver burden (according to ZBI).

Biomarker	Without Burden (*n* = 62)	With Burden (*n* = 16)	Mann–Whitney U Statistic	*p*-Value
Cortisol (ng/mL)	48.03 (35.64–68.64)	43.64 (23.10–55.47)	393.00	0.202
TNF-α (pg/mL)	2.36 (0.174–7.48)	1.00 (0.174–2.90)	368.50	0.112
IL-6 (pg/mL)	18.14 (10.86–28.62)	23.78 (17.07–47.25)	374.50	0.133
CRP (mg/L)	1.55 (0.68–3.91)	2.85 (0.87–4.12)	441.00	0.496

Note. Values are presented as median (interquartile range: 25th–75th percentile). Normality was assessed using the Shapiro–Wilk test, revealing a non-normal distribution. Comparisons were made using the Mann–Whitney U test. Abbreviations: ZBI, Zarit Burden Interview; TNF-α, Tumor necrosis factor alpha; IL-6, Interleukin-6; CRP, C-reactive Protein; ng/mL, nanograms per milliliter; (pg/mL), picograms per milliliter; mg/L, milligrams per liter.

## Data Availability

The data used for this article are available as Supplementary Material. If additional information is required, please contact the corresponding author.

## Supplementary Materials

The following supporting information can be downloaded at: https://www.mdpi.com/article/10.3390/ijms27073316/s1.
